# Evaluation of Sympathetic Skin Response in Old-Polio Patients

**Published:** 2011-11-01

**Authors:** M R Emad, K Pakmanesh, P Sedaghat

**Affiliations:** 1 Department of Physical Medicine and Rehabilitation, Shiraz University of Medical Sciences, Shiraz, Iran

**Keywords:** Poliomyelitis, Autonomic neuropathy, Sympathetic nervous system

## Abstract

**Background:**

Many polio patients develop problems such as cold intolerance in the affected limbs, which seems to be due to sympathetic nervous system dysfunction. This study aimed to investigate whether there is a sympathetic system dysfunction in old-polio patients by means of the systematic skin response test.

**Method:**

Forty old-polio patients and 20 healthy subjects were included in the study. Disease duration was 31.5 years (19-49 years) in the patient group. Sympathetic skin responses were obtained in all the subjects’ limbs. Thirteen patients had right lower limb paresis/paralysis, 14 had left limb paresis/paralysis and 13 had paresis/paralysis of both lower limbs. The upper limbs were unaffected in all the patients.

**Results:**

Although there was no significant difference between sympathetic skin response latencies of the case and control groups, the amplitude values of the sympathetic skin response in the patient’s lower extremities were significantly lower than those in the control group.

**Conclusion:**

There was a sympathetic nervous system dysfunction in some old-polio patients. This finding might be useful in evaluation and treatment of old-polio patients, developing new problems.

## Introduction

MTPoliomyelitis is a viral disease resulting from polio viruses which in acute stages involves both motor neurons of the spinal cord and autonomic nervous system. Involvement of this system results in different degrees of increase in the blood pressure and increase or decrease of sweating in the affected segments.[1] There is little information regarding the persistence of dysfunction I nautonomous nervous system in old-polio patients and researchers are not in agreement in this field.[2][3]

Many patients reported symptoms such as cold intolerance which could be attributed to sympathetic system disorder.[3] Sympathetic skin response test (SSR) is simple, quick, easy and non-invasive method which is often used in neurophysiology laboratories to examine the sympathetic system. This response results from synchronic activation of eccentric sweet glands, due to sympathetic afferent fibers activity.[4][5]

SSR could be obtained with both internal stimulation as coughing, sneezing, deep breathing and peripheral nerve stimulation.[6][7] This research was done in order to examine the sympathetic system in old-polio patients by means of sympathetic skin response test.

## Materials and Methods

Forty old-polio patients (12 males and 28 females) with a mean age of 33.7 years (20-28 years) and 20 healthy subjects (10 males and 10 females) with a mean age of 34.9 years (21-47 years) were included in the study. Disease duration was 31.5 years (19-49 years) in the patient group.

Patient with diabetes, peripheral neuropathy, peripheral nerve damage and any other disease which could affect the sympathetic nervous system and SSR test and also all the individuals who used medicines which affected the sympathetic nerve system and SSR test were excluded from this study. All the candidates signed the informed consent form for participation in the research and all the information were confidential. Manual muscle testing was used to examine the muscle power and determine the involved limbs. Muscle groups to be checked up were flexor and extensors of the foot and hand fingers, wrist and ankle, knee, elbow shoulder, hip, elbow supinator and pronator of the elbow abductor of the shoulder and hip.

SSR test was recorded by electromyograph device (Tonnies Multilinear, version 2.0 Electromygraph) with disk surface electrodes using sweep speed of 500 ms/div, sensitivity of 500-1000 Mic.v/div, filtering of

0.5 hz-2 khz and frequency of stimulation equal to 1 stimulus/20-30 sec. The study was conducted under standard condition, in a semi dark and quiet room at temperature of 22 to 24 centigrade. The skin temperature was maintained above 32 centigrade. For recording the median nerve SSR, the active electrode was placed on the base of the second finger on the palmer surface of the hand, reference electrode on the dorsum of the hand, grand electrode on the wrist and stimulator electrode on the wrist between palmaris lounges and flexor carpi radialis.

To record SSR from the tibial nerve, the active electrode was placed on the sole of the foot with 3cm distance from first and the second finger, the reference electrode was placed on the dorsum of the foot and the grand electrode on the fifth metatarsus base on the sole of the foot. Also, the stimulator was placed on the tibial nerve on the back of the medial maleolluls. The stimulation was begun with 5 milliamper intensity and increased up to 30 milli-amper, if necessary. In order to avoid habituation, a 20-30 seconds pause between successive stimuli was considered. Wave latency was considered from the stimulus artifact to the beginning of the wave and the wave amplitude was calculated from the negative peak to positive one. To statistically analyze the data, t-test was used and p<0.05 was considered significant.

## Results 

According to Manual Muscle Test (MMT), none of the patients had problem in their upper extremities. Of the 80 lower examined limbs in the patients group, 35 limbs had problem (muscular weakness and atrophy) and 27 did not. Except one affected foot with no SSR response, the responses were recorded for all the other limbs.

There was no significant difference between SSR latencies recorded from the upper and lower extremities of the patients when compared to the control group. The mean SSR amplitude recorded from the patient's hands was less than that of the control group, but their difference was not statistically significant. The mean amplitude of SSR recorded from the patients feet was less than that of the control group, and their difference was statistically significant (p=0.001; Table 1 and Table 2).

**Table 1:** Latency and SSR Amplitude in the upper extremities of old poliomyelitis patients and the control group

	**Poliomyelitis patients**	**No.**	**Mean+SD**	****	**No.**	**Mean+SD**	***P *value**
Latency (Second)	Right hand	40	1.41+0.12	Control	40	1.42+0.13	0.810
	Left hand	40	1.42+0.11				0.910
Amplitude (Millivolt)	Right hand	40	1.3+0.46	Control	40	1.53+0.38	0.058
	Left hand	40	1.37+0.64				0.084

**Table 2:** Latency and SSR^a^ Amplitude in the lower extremities of the old poliomyelitis patients and the control group

	**Poliomyelitis patients**	**n**	**Mean+SD**	****	**n**	**Mean+SD**	***P *value**
Latency (Second)	Affected side	52	1.96+0.18	Control	40	1.95±0.16	0.690
	Non-affected side	27	1.93+0.13				0.710
Amplitude (Millivolt)	Affected side	52	0.66+0.29	Control	40	1.25+0.34	0.001
	Non-affected side	27	1+0.36				0.036

^a^ SSR was absent in one affected foot.

## Discussion

To the best of our knowledge, no study has been done on SSR electro-diagnosis test in old poliomyelitis patients so far. Therefore, no study was found so that we could compare with the present study. Little information is available in medical texts regarding autonomous system dysfunction in old poliomyelitis patients. Examining cardiovascular autonomous responses in 20 old-polio patients, Brog and colleagues concluded that there was no obvious autonomic nervous system (sympathetic and parasympathetic) dysfunction.[2] On the other hand, Bruno et al. believed that these patients had sympathetic system dysfunction and this, resulted in a sympathetic output decline to skin veins, dilation of the surface veins, and as a result blood stasis which resulted in heat loss and coldness of the limbs, as commonly seen in these patients.[3]

In this study, the mean amplitude of SSR recorded from the patient's feet (affected and non-affected) was less than that of the control group. The decrease in the amplitude was more in their affected feet than in the non-affected ones. SSR amplitude decline could be a reason for sympathetic system dysfunction in the examined patients because in some studies SSR amplitude decline was known as a reason for sympathetic system dysfunction.[8] It is hard to judge about the place and origin of the injury that caused a decline in SSR amplitude, but due to the amplitude decline recorded from the weak feet, injury to the distal part of SSR afferent nerves especially in the lateral horn of the spinal cord seems to be the reason.

In conclusion, the results of this study suggest that there is sympathetic nervous system dysfunction in some old-polio patients. This finding might be useful in evaluation and treatment of old-polio patients, developing new problems. Nevertheless, more studies with larger sample size and patients with affected upper limbs is required so that more accurate conclusions regarding the sympathetic skin response in these patients could be achieved.
